# Prevalence of advance care planning practices among people with chronic diseases in hospital and community settings: a retrospective medical record audit

**DOI:** 10.1186/s12913-021-06265-y

**Published:** 2021-04-05

**Authors:** Sarah Jeong, Tomiko Barrett, Se Ok Ohr, Peter Cleasby, Ryan Davey, Michael David

**Affiliations:** 1 School of Nursing and Midwifery, University of Newcastle, 10 Chittaway Road, Ourimbah, NSW 2258 Australia; 2 Department of Aged Care Services, Wyong Hospital, Central Coast Local Health District, PO Box 4200, Lakehaven, NSW 2263 Australia; 3 Hunter New England Nursing and Midwifery Research Centre, Hunter New England Local Health District, James Fletcher Campus, Gate Cottage, 72 Watt St, Newcastle, NSW 2300 Australia; 4 Division of Aged, Subacute and Complex Care, Central Coast Local Health District, PO Box 6088, Long Jetty, NSW 2261 Australia; 5 School of Nursing and Midwifery, University of Newcastle, 10 Chittaway Road, Ourimbah, NSW 2258 Australia; 6 School of Medicine and Public Health, University of Newcastle, Callaghan, NSW 2308 Australia

**Keywords:** Advance care directive, Advance care planning, Chronic disease, Community, Hospital, Clinical trial

## Abstract

**Background:**

Advance Care Planning (ACP) enables healthcare professionals to embrace the important process where patients think about their values in life and goals for health care, and discuss their future health care preferences with family members for a time when they are not able to make health care decisions. Despite the promotion of ACP last two decades, and well-known benefits of ACP and a written Advance Care Directive (ACD), they are still underutilised in Australia and across the world. Previous studies have provided some insights, however, an uptake of ACP and prevalence of ACDs in community settings is rarely reported.

**Methods:**

The aim of this study was to determine the uptake of ACP and prevalence of ACDs among people with chronic diseases in hospital and community settings. A retrospective medical record audit of eligible patients looking for evidence of ACP was conducted in 16 research sites in eight hospital and eight community care settings. Participants included those who were admitted to one of the research sites, and who were aged 18 years and over with at least one of nine nominated chronic diseases. The primary outcome measures included the number of patients with evidence of ACP through the following practices: completion of an ACD, appointment of an Enduring Guardian (EG), or completion of a resuscitation plan.

**Results:**

The overall prevalence of ACD was 2.8% (*n* = 28) out of 1006 audited records, and only 10 (1%) of them were legally binding. The number of EGs appointed was 39 (3.9%) across the sites. A total of 151 (15.4%) resuscitation plans were found across the eight hospital sites. 95% (*n* = 144) of the resuscitation plans indicated ‘Not-for-resuscitation’.

**Conclusions:**

The uptake of ACP is very low. Current medical recording system reveals the challenges in ACP lie in the process of storage, access and execution of the ACDs. Given that having an ACD or Enduring Guardian in place is only useful if the treating physician knows how and where to access the information, it has implications for policy, information system, and healthcare professionals’ education.

**Trial registration:**

The study was retrospectively registered with the Australian New Zealand Clinical Trials Registry (Trial ID: ACTRN12618001627246). The URL of the trial registry record http://www.anzctr.org.au/trial/MyTrial.aspx

**Supplementary Information:**

The online version contains supplementary material available at 10.1186/s12913-021-06265-y.

## Background

Chronic disease contributes to more than 70% of the disease burden in Australia, with 87% of older Australians living with at least one chronic condition such as cancer, cardiovascular disease and diabetes [1, 2]. People with chronic diseases represent 24% of total hospitalisations and 75% were emergency admissions. Although about 42% of this sub-cohort died in hospital, only 4% of them received palliative care services [3, 4] despite the evidence that these patients will benefit from such care for a considerable period prior to their death [5]. The concept of Advance Care Directive (ACDs), was first proposed by Kutner in 1967 [6] to improve end-of-life care, and was adopted by the United States (US) government in 1991 [6, 7]. ACDs were acknowledged as legally binding in Belgium (2002), Denmark (2008), France (2005), Germany (2009), The Netherlands (1995), Spain (2002), the United Kingdom (UK) (2005) and Switzerland (2008) [8]. Since then there has been growing interest and research to promote ACDs around the world. However, documentation rates of ACDs have been low to very low for three decades worldwide [7, 9–11]. Extensive research conducted across the world has led to the conclusion that the ACDs, as simply ‘statements made by an individual’, were neither understood nor accepted by individuals or healthcare professionals [10–12]. Hence, Advance Care Planning (ACP) has emerged as a response to this low uptake and practice. ACP enables health care professionals to embrace the important process where patients think about their values in life and goals for health care, and discuss their future health care preferences with family members for a time when they are not able to make health care decisions [7, 9, 13, 14].

Various terms appear to describe the need to make decisions for ‘end-of-life’ care in the literature. Nevertheless, there is a shared understanding of ACDs as a written form of directives or statements for future treatment preferences and wishes. However, internationally, for ACP as a process there is a lack of consensus on what ACP entails and how to define successful ACP [15]. To fill this gap, Sudore et al. [15] constructed outcome measures for successful ACP by a Delphi panel of 52 multidisciplinary and interventional ACP experts, and reported ‘care consistent with goals’ as the top-rated outcome measure. In another research, a five-round Delphi study supported by the European Association for palliative care was conducted to define ACP [16]. Fifteen recognised experts from eight countries (Belgium, Canada, Germany, Ireland, Italy, Netherlands, UK, USA) reached a consensus that ACP includes the documentation of preferences and the appointment of a proxy decision maker. Similarly, in New South Wales (NSW), Australia, according to the Ministry of Health [17], outcomes from ACP can include a written ACD and an appointment of a legally binding substitute decision-maker. Although there is no legislation providing for ACDs in NSW, ACDs are legally binding under the Common law if it is completed by a legally competent adult [18]. Theoretically, it is ideal if ACDs are documented as a result of ACP, although individuals may decline to do an ACD [17]. Anecdotal evidence suggests that ACP may not result in legally binding ACDs documented by a competent adult but may result in ‘other ACP documentation’ such as resuscitation orders, or nomination of a next-of-kin(s), that has no legal authority to make decisions over personal, medical and lifestyle matters [17], and/or personally written letter to direct care [14].

Even after reaching the agreement that ACP involves an appointment of a substitute decision-maker, the complexity of defining ACP also lies in the various terms around substitute decision-maker in Australia and worldwide. A formally appointed substitute decision-maker is called a ‘Lasting Power of Attorney for Health and Welfare’ in the UK [19], and ‘a legal proxy’ in German [20], whilst in Australia, this is known as an ‘Enduring Power of attorney’ in Australian Capital Territory or ‘medical treatment decision-maker’ in Victoria [18]. In NSW, a formally appointed substitute decision-maker is called an Enduring Guardian by the NSW Guardianship Act 1987 [17, 18]. The challenges with the lack of uniform definition and clarity of the concept include misunderstanding of the core concept of ACP, underreporting or misreporting of the prevalence of ACP. More importantly, it is difficult to share the learnings from various studies globally, and for governments and health service providers to monitor ACP policy and effectiveness of interventions [14].

ACP has been promoted over the last two decades. Although a recent trial reported that the ACP intervention facilitated by trained nurses and allied health professionals did not make difference in patient and family satisfaction with care [21], the benefits of ACP are well-known including increased autonomy and reduced burden of decision-making [7, 9]. However, they are still underutilised in the Australian health care system [17, 21] and across the world [19–25]. In a Statewide-population survey [26] (*n* = 3055) in South Australia, more respondents reported having completed the enduring power of attorney (22%) for financial decisions than any of the health care-related documents — enduring power of guardianship (13%), medical power of attorney (11%), and anticipatory direction (12%). A recent multicentre audit study in Australia [14] reported that ACD prevalence was significantly higher in residential aged care facilities (formerly known as nursing homes) (47.7%) but was still low in hospitals (15.7%) and general practices (3.2%) (*p* < 0.001) and varied across jurisdictions.

Previous studies that investigated the uptake of ACP and/or prevalence of ACDs have provided some insights. However, it must be noted that; 1) the terminology and documentation requirements vary locally, nationally and internationally, 2) previous attempts have relied on self-reporting [26], and 3) settings have been limited to institutions such as hospitals, residential aged care facilities or general practices [7, 9, 11, 14, 27]. An uptake of ACP and/or prevalence of ACDs in community setting is not widely reported [22]. More importantly, given the various terms, legislation frameworks, requirements, resources, forms and services available across Australia, the importance of assessing the prevalence of ACP within the local context is paramount for policy and service development, but is rarely reported.

The aim of the current study was to examine the prevalence of ACP in two Local Health Districts (LHDs) in NSW, Australia prior to a trial of normalised ACP service which was implemented for people with chronic conditions in hospital and community settings. The trial was a quasi-experimental study and the study protocol is reported elsewhere [28]. For this study, the chronic diseases included cancer, chronic kidney disease, chronic obstructive pulmonary disease, congestive heart failure, coronary artery disease, dementia, diabetes, frailty and hypertension and are aligned with special ACP needs in the NSW Action Plan 2013–2018 [13].

## Methods

A retrospective medical record audit of eligible patients looking for evidence of ACP was conducted in 2018. This project was approved by the Hunter New England Human Research Ethics Committee (Approval No. 17/12/13/4.16). A total of 16 sites across two LHDs (eight in each) that covered both hospital and community settings, and both government and non-government agencies were selected. Sites from two LHDs were invited to participate after obtaining the Ethics approval (Approval number: 17/12/13/4.16). Participating sites were eight wards in acute hospitals, which were pair-matched based on admission rates, patient characteristics, average length of stays, number of deaths per month/year, and number of referrals from/to hospital and community. The eight community settings included four public and four non-public home and community care service providers.

Medical records of patients/clients were requested from relevant medical records departments and the inclusion criteria included those; 1) who were admitted to one of the 16 research sites in the timeframe between April and May 2018, 2) who were aged 18 and over, and 3) who had at least one of the chronic conditions enumerated as above. It should be noted that the term admission includes service requests/home visits for patients receiving care from hospital or community health nurses. Records were audited for the evidence of ACP. For the purpose of this study the evidence of ACP included the following.

1. ACD: a legally binding document made by a legally capable person about the person’s specific wishes and preferences for future care. This includes treatments they would accept or refuse if they had a life-threatening illness or injury, their values in life and goals of care [17]. For an ACD to have sufficient authority to act on, the four standards should be satisfied including specificity, currency, competence and witnessing [29, 30].
2. An Enduring Guardian: an individual(s) who is legally appointed by the person and who can legally make decisions on behalf of the person about the person’s medical and dental care, if the person loses capacity to make decision [17].

In NSW, ‘a resuscitation plan’ guides medical and healthcare professionals in using or withholding resuscitation measures and other aspects of treatment relevant at end of life, and is legally binding [31]. Resuscitation plans are written by medical officers in hospitals and do not consistently include a discussion with the patient. For this reason, it does not meet the definition of ACP in NSW but it is a common practice in hospital settings. Hence, the presence of ‘resuscitation plans’ was captured but not included in the overall prevalence of ACP.

Audits were conducted by two trained research assistants using a pilot-tested and standardised approach. The method used to search for evidence of ACP varied on the type of medical records kept to paper or electronic. All identified ACDs were reviewed for validity according to NSW jurisdiction. The detailed audit process is provided in Additional file 1.

All analyses were performed using Stata Version 15.0 (StataCorp LP, College Station, TX, USA). Due to the extremely low evidence of ACP, multi-level regression analysis planned in the study protocol [28] was not conducted but descriptive statistics were used to summarise the data as recommended by a statistician.

## Results

### Demographic characteristics of patients

In total 1006 patients’ medical records were audited with 529 records in LHD-1 and 477 records in LHD-2. Demographic characteristics are presented in Table 1. In total, 47% of patients were male and 53% were female. The mean age of patients was 77 years in LHD-1 and 74 years in LHD-2. The mean age of patients was 76 years in hospitals and 75 years in community sites. Diabetes (60%) and Hypertension (53%) were the two most common chronic conditions reported.

**Table 1:** Demographic characteristics of participants

	LHD1 (***n*** = 529)		LHD2 (***n*** = 477)		Total (***n*** = 1006)
	Hospital	Community	Hospital	Community	
N	291	238	159	318	1006
Mean age (SD)	76 (14.36)	78 (11.66)	75 (13.26)	72 (15.82)	75 (2.5)
Sex (M:F) %	48: 52	46: 54	50: 50	45: 55	47: 53
Chronic conditions					
Diabetes	152 (55)	125 (45)	107 (32)	223 (68)	607 (60%)
Hypertension	169 (65)	91 (35)	88 (32)	188 (68)	536 (53%)

Table 1 Legend-*LHD* Local Health District, *SD* Standard Deviation, *M* Male, *F* Female

### Prevalence of ACP

All audit results are based on the medical notes (electronic and/or paper based) and all the ACD documents found are summarised in Table 2. There were a low number of ACDs found across all sites. The overall prevalence of ACD was 2.8% (*n* = 28) out of 1006 audited records with a slightly higher prevalence of 4.7% (*n* = 25) in LHD-1, compared to 0.6% (*n* = 3) in LHD-2. Out of 28 ACDs found, nine (1.7%) of ACDs in LHD-1 and only one (0.2%) in LHD-2 were legally binding. 16 (3.0%) and two (0.4%) ACDs in LHD-1 and LHD-2 respectively were legally nonbinding. Those nonbinding ACDs were due to being signed by ‘Power of Attorney’ who does not have a legal authority to make medical and lifestyle decisions in NSW. Two other patients who were transferred from a residential aged care facility had an ACD which was signed by ‘Power of Attorney for Health Care’ which is not a legalised term nor has a legal authority to make medical and lifestyle decisions in NSW. There was one case of staff referring to ‘resuscitation plan’ as ACDs. In another case, there was a written note by a social worker that “patient has come to terms with end of life/ ACP was discussed and documented”, but ACD was not located in the medical record. The prevalence of legally binding ACDs in hospital setting was 1.3% (*n* = 7) in LHD-1 and 0.2% (*n* = 1) in LHD-2. The prevalence of ACDs in community setting in LHD-1 was 0.4% (*n* = 2) and no ACD was found in community setting in LHD-2. LHD-2 Hospital site 3 and 4 were more closely examined as it was the only hospital sites to show no evidence of ACDs. On admission, nurses routinely fill out an ‘Adult inpatient admission and risk assessment’ form electronically. This form includes a check box asking if the patient does have a pre-existing adult resuscitation plan/ACD/ACP. In 17 of 82 patients (21%) this was ticked yes but there was no evidence of the ACP/ACD in any other part of the patient’s record. Twenty-three (28%) of the ‘Adult inpatient admission and risk assessment’ forms were either missing or blank.

**Table 2:** Prevalence of ACDs, Enduring Guardians and Resuscitation plan

Site (***n*** = 1006)	Number of ACDs		Number of EGs	Resuscitation Plan (NFR)	Resuscitation Plan (FR)
	Legally binding	Legally nonbinding			
**LHD-1 (*n*** **= 529)**					
Hospital 1 (*n* = 65)	0	2	1	3	0
Hospital 2 (*n* = 75)	3	7	5	16	0
Hospital 3 (*n* = 74)	3	1	1	34	1
Hospital 4 (*n* = 77)	1	6	2	17	2
Community 1 (*n* = 58)	1	0	1	NA	NA
Community 2 (*n* = 104)	0	0	0	NA	NA
Community 3 (*n* = 73)	1	0	2	NA	NA
Community 4 (*n* = 3)	0	0	0	NA	NA
**Total**	**9 (1.7%)**	**16 (3.0%)**	**12 (2.3%)**	**70 (24%)**	**3 (1.0%)**
**LHD-2 (*n*** **= 477)**					
Hospital 1 (*n* = 29)	0	1	4	10	0
Hospital 2 (*n* = 48)	1	1	13	23	0
Hospital 3 (*n* = 20)	0	0	0	16	2
Hospital 4 (*n* = 62)	0	0	1	25	2
Community 1 (*n* = 131)	0	0	9	NA	NA
Community 2 (*n* = 77)	0	0	0	NA	NA
Community 3 (*n* = 106)	0	0	0	NA	NA
Community 4 (*n* = 4)	0	0	0	NA	NA
**Total**	**1 (0.2%)**	**2 (0.4%)**	**27 (5.7%)**	**74 (47%)**	**4 (2.5%)**

Table 2 Legend-*LHD* Local Health District, *ACDs* Advance Care Directives, *EGs* Enduring Guardians, *NFR* Not For Resuscitation, *FR* For Resuscitation

### Prevalence of enduring guardians

The number of Enduring Guardians legally appointed was 39 (3.9%) across the sites, with prevalence of 2.3% (*n* = 12) in LHD-1 and 5.7% (*n* = 27) in LHD-2. The prevalence of Enduring Guardian appointment in hospital setting in LHD-1 was 1.7% (*n* = 9) and was 3.8% (*n* = 18) in LHD-2. The prevalence of Enduring Guardian appointment in community setting in LHD-1 was 0.6% (*n* = 3) and 1.9% (*n* = 9) in LHD-2.

**Fig. 1:**
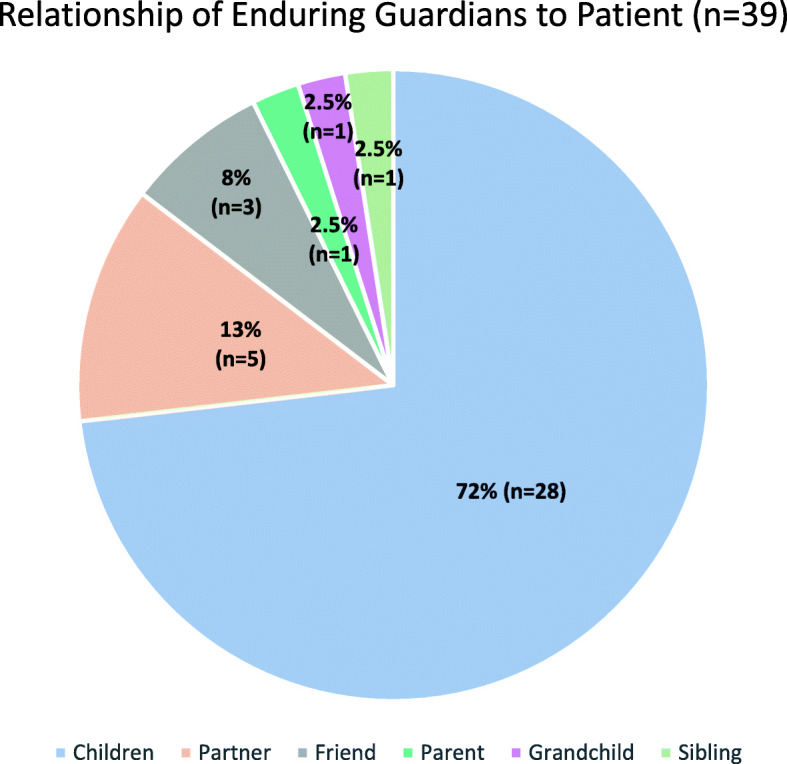
Relationship of Enduring Guardians to patient

The relationship of the Enduring Guardian to the patient is presented in Fig. 1. Of those (*n* = 39) who appointed legally valid Enduring Guardian, 72% (*n* = 28) elected their child as their Enduring Guardian, and nine people nominated two children (e.g. daughters, sons or daughter and son) as their substitute decision-makers. It should be noted that this included stepchildren and children-in-law, although this was a small fraction of the cases.

### Prevalence of resuscitation plan

A total of 151 resuscitation plans were found from a total of 450 audited medical records across the eight hospital sites. The prevalence of resuscitation plans was 25% (73 of 291) in LHD-1 and 49% (78 of 159) in LHD-2. 95% (*n* = 144) of the resuscitation plans indicated ‘Not-for-resuscitation’. One in five (21.2%, *n* = 32 of 151) of the resuscitation plans found were not signed by a healthcare professional. It should be noted that incomplete resuscitation plans were still recorded in the ‘Not For Resuscitation (NFR)’ and ‘For Resuscitation (FR)’ columns although an incomplete plan is not a legally binding document in NSW.

## Discussion

### Low prevalence of ACDs and EGs

Given the lack of available evidence on the practice of ACP in community setting, this study adds new insights about the prevalence of ACP in both government and non-government community settings. Like previous studies [14, 22], a low number of ACDs and Enduring Guardians were found in patient records. Only 10 (1%) of the patients had a legally binding ACD and 39 (3.9%) of the patients had an Enduring Guardian. It is beyond the scope of this study and extensive research [7, 9, 13, 14, 22] already suggests that the reasons for low awareness of ACD and appointment of Enduring Guardian include; ‘Don’t know how to do it’, ‘Difficult to understand the form’ ‘Difficulty to understand what it is’, ‘Don’t want to upset my family’, and ‘Time consuming’. Despite the various reasons, one consensus is that the onus is on health professionals to initiate ACP to assist patients [32]. It is timely that a recent systematic review [33] suggested that there are preconditions in multiple domains at micro, meso and macro levels to implement ACP successfully and as such it requires a whole-system approach. The findings of this retrospective audit will inform the main study [28] which embedded multiple mechanisms to implement a normalised ACP service.

### Resuscitation plans

There was a much greater number of resuscitation plans recorded in patient records compared to both Enduring Guardian documentation and ACD. One concern with resuscitation plans found in the audit was that 32 of the total 151 were not signed by a medical officer. This is troubling as it calls into question the legal satus of the document should a doctor have to decide about whether to attempt resuscitation. Another question is why doctors initiate and complete more resuscitation plans than ACDs. Discussion and completion of a resuscitation plan should prompt discussion for completion of an ACD. The fact that a resuscitation plan does not necessarily need to involve the patient, but can be written for the person, whilst ACDs can be only completed by the individual themselves [17], may explain one aspect. The exact reasons for the more prevalent use of resuscitation plan than ACD warrant a further investigation.

### Inconsistency and inaccessibility of location and storage of ACDs and enduring Guardian appointment

The process of auditing revealed that the current medical record system used to record patient information are either ill-equipped or underutilised to record the presence of an ACD. For LHD-2, most of the records were searched using ‘PowerChart’ for hospital patients (See Supplementary file 1). This system was the only one to specifically have an ‘Advanced Care Planning’ tab in an obvious and accessible location. This made it easier to find ACDs in the auditing procedure. It would also make it easier for treating healthcare professionals to access when necessary. Yet five out of nine ACDs found in LHD-2 were all located by manually searching through patients’ paper medical records. Treating health care professionals in the Emergency Department are unlikely to have access to the paper record in subsequent admissions. On the other hand, LHD-1 used medical record access programs such as Digital Medical Record (DMR) and Community Health Information Management Enterprise (CHIME), where the recording of ACDs was far more inconsistent. This made it difficult to find evidence of ACP, which means a greater chance of reporting a false negative for their patients.

The auditing also revealed inconsistent or inappropriate recordings by healthcare professionals in ‘Adult inpatient admissions and risk assessment form’. Inconsistent recording was also evident in EG, as there were numerous mentions of Enduring Guardians in patient notes with no formal documentation found. This was evident in patient records at all sites, but in particular, in community sites.

Various types of ACD documents have been reported to be a challenge to compare and evaluate effectiveness of ACP interventions internationally [14, 30]. In this study, we add new evidence to extend that the challenge also lies in the storage of ACP related documents which was inconsistent and did not facilitate timely access to or execution of these documents. One study by Cheang et al. [34] revealed in 2014 that out of 100 patients, there was no record of ACDs in their medical files, despite 12% of patients reporting an ACD in place in their interview. Despite the fact that this has a serious implication for clinical practice, policy and systems to ensure easily accessible storage to be available for all involved, it still remains an issue and warrants attention. The launch of the national digital health record system ‘My Health Record’ in Australia offers a potential solution for all to store and access ACP practices and ACDs documented across care settings. However, given that some individuals may opt out, alternative and better medical record systems are necessary to capture ACP activity. It also has implications for treating healthcare professionals’ education regarding accurate and comprehensive collection of ACP information, followed by consistent storage in a designated, accessible location.

### Limitations

The study is limited as it relied on medical records only although the rigour and validity were ensured as in the detailed audit process (Additional file 1). Therefore, ACP/ACD or Enduring Guardian documentation may have been held by the patient, general practitioner, specialist or other healthcare professional were not captured in this audit. However, the methodological strengths include that the study was conducted at multi-centre across two health districts with the adequate sample size and clear inclusion criteria. The findings of the audit may be limited to the 16 study sites with only a small number of evidences of ACP found, hence should be interpreted with caution.

## Conclusion

International literature suggests that a consensus on the definition of ACP and understanding how to measure successful ACP are fundamental for policy makers and health service providers to monitor ACP policy and effectiveness of interventions, but it has been a challenge globally due to the lack of uniform definition and clarity of what entails ACP. This retrospective medical record audit was conducted to examine the prevalence of ACP within the local definition and legislative framework in NSW, Australia. Despite the policy, legislative framework and resources available to promote ACP, its prevalence remains very low in both hospital and community settings in NSW. In addition to this, healthcare professionals record keeping reflects a poor understanding and possible lack of commitment, and the current medical record systems appear ill-equipped to correctly record ACP practices and ACD documents in real time. Having an ACD or Enduring Guardian in place is only useful if the treating healthcare professionals know where and how to access the information. To ensure the benefits for all involved, both patients and healthcare professionals need to be educated, and ACP needs to be promoted with a whole-system approach. This may become plausible and feasible with the normalisation of ACP service provision supported by enhanced processes and system to store, locate, access and execute ACP practices and ACD documents.

## Supplementary Information

**Additional file 1.** Auditing Method.

## Data Availability

All data generated or analysed during the pre-intervention period are included in this published article and its supplementary information file.

## Abbreviations

ACD: Advance Care Directives
ACP: Advance Care Planning
FR: For-Resuscitation
LHD: Local Health District
NFR: Not-For-Resuscitation
NSW: New South Wales
UK: The United Kingdom
