# Supplementary material for: Prevalence of advance care planning practices among people with chronic diseases in hospital and community settings: a retrospective medical record audit
Source: BMC Health Serv Res. 2021 Apr 5;21:303. doi: 10.1186/s12913-021-06265-y (PMC8022421; doi:10.1186/s12913-021-06265-y)
Supplement: Supplementary file 1 — Additional file 1. Auditing Method. [file 12913_2021_6265_MOESM1_ESM.docx]

**Additional file 1. Auditing Method**

Table 1 shows a summary of medical record audit to identify the evidence of the ACP and ACD. The medical record audits for all 12 public sites were conducted either electronically or manually by the two research assistants and the research team according to the Standard procedure of conducting the pre-audit for NACP Study. At four non-public sites, they conducted their own internal pre-audit as per organisation’s obligation to comply with ‘Confidentiality and Privacy’ legislation and policy. The Care Coordinators of those sites were trained about the evidence of ACP and data collection tool.

Table 1. Summary of Medical Record Access tools used to complete audit.

| **Site No.** | **Description** | **Medical Record Access** |
| --- | --- | --- |
| LHD-1-01 | Hospital – Government Site | Electronic - PowerChart |
| LHD-1-02 | Hospital – Government Site | Electronic - PowerChart |
| LHD-1-03 | Hospital – Government Site | Electronic - PowerChart |
| LHD-1-04 | Hospital – Government Site | Electronic - PowerChart |
| LHD-1-05 | Community Health Care – Government Site | Electronic - PowerChart |
| LHD-1-06 | Community Health Care – Non-Government Site | Internal Audit |
| LHD-1-07 | Community Health Care – Government Site | Electronic - PowerChart |
| LHD-1-08 | Community Health Care – Non-Government Site | Internal Audit |
| LHD-2-01 | Hospital – Government Site | Paper record search |
| LHD-2-02 | Hospital – Government Site | Paper record search |
| LHD-2-03 | Hospital – Government Site | Electronic - DMR |
| LHD-2-04 | Hospital – Government Site | Electronic - DMR |
| LHD-2-05 | Community Health Care – Government Site | Electronic - CHIME |
| LHD-2-06 | Community Health Care – Non-Government Site | Internal Audit |
| LHD-2-07 | Community Health Care – Government Site | Electronic - CHIME |
| LHD-2-08 | Community Health Care – Non-Government Site | Internal Audit |

**Evidence of Advance Care Planning**

Advance Care Planning could include one or more of the following:

1. Advance Care Directive (ACD): a legally binding document made by a legally capable person about the person’s specific wishes and preferences for future care. This includes treatments they would accept or refuse if they had a life-threatening illness or injury, their values in life and goals of care^17^. For an ACD to have sufficient authority to act on, the four standards should be satisfied including specificity, currency, competence and witnessing^18,19^.

2. Enduring Guardian (EG): an individual(s) who is legally appointed by the person and who can legally make decisions on behalf of the person about the person’s medical and dental care, if the person loses capacity to make decision^17^.

3. Details of the person the client would like to make medical decisions on their behalf, if they lose the ability to make decisions.

4. Any records or notes relating to ACP: This could include details of what is important to the client; values, life goals and preferred outcomes. Also the treatments and care they would like or would refuse if they had a life-threatening illness or injury.

5. Documented conversations, between an individual, their family, carer &/or health professional regarding their future health care preferences.

6. Resuscitation plans or 'medical orders' generated with the client (including those limiting treatment, such as 'not for CPR', 'not for intubation', 'not for intensive care unit (ICU)', 'not for hospitalisation' or 'not for antibiotics').

**Sources of the medical audits**

**1. Paper based medical record audit**

For paper records, which were used in two of the 16 sites, the search strategy was simple. The auditor would manually examine the medical records of each eligible patient’s admission. The admissions during the audit period of April – May 2018 were examined for any evidence of the ACP, which are ACDs and EG.

**2. PowerChart**

The following protocol was used to look for evidence of ACP in PowerChart.

1. Check ‘advanced care planning’ tab to look for any scanned ACD/ACP documents and resuscitation plans.
2. Check in ‘documentation’ for patient admission notes. Use filter to search for the following types of documents within the date range of the patient’s admission.

- Advanced Care Planning
- Enduring Guardian
- Legal
- Resuscitation plans (for acute care site auditing).

**3. Community Health Information Management Enterprise (CHIME)**

The following protocol was used to look for evidence of ACP in CHIME.

1. Check ‘client info’, ‘alerts’ section for any reference to ACP
2. Check ‘client info’, ‘personal’ section for EG (legal).
3. Go to: ‘Reports’, ‘select’ under ‘client’ and ‘service request’ – select *all* services and select all parameters, including ‘clinical notes’ to generate a report
4. Use ‘ctrl + F’ and search for the following **search terms**:
   - Advance (covers care plan and directive)
   - ACP
   - ACD
   - Living will
   - Maker (covers SDM and medical decision maker)
   - Substitute
   - Enduring (for EG)
   - SDM
   - Future
   - Resus (for resuscitation)
   - Refus (for refuse or refusal)
   - Wishes
   - Discuss

**4. Digital medical records (DMR)**

The following protocol was used to look for evidence of ACP in DMR. Although electronic, DMR used handwritten notes scanned in to their database, so using the ‘ctrl +F’ search terms were not applicable.

1. Search through admissions tab for relevant stay.
2. Search through admission notes for copies of, or references to, the following documents.

- Advanced Care Directives
- Enduring Guardian Forms
- Resuscitation Plans

1. Check in ‘Medico-Legal’ tab for any supporting documentation.

**Information recorded**

For both electronic and paper records, where evidence was found in one of the above practices, the details were recorded to answer the following questions.

- Does the patient have an Advanced Care Directive in their records?
- What section of the report mentions the presence of an Advanced Care Directive?
- Is the ACD found complete and/or legally binding?
- Does the patient have a legally appointed EG? If so, has the documentation been sighted?
- What is the relationship of the EG to the patient?
- For the hospital settings, did the patient have a resuscitation plan?
- Did the patient wish or not wish to undergo CPR?
- Is the resuscitation plan found complete and/or legally valid?
